# Supplementary material for: Abundant Species Diversity and Essential Functions of Bacterial Communities Associated with Dinoflagellates as Revealed from Metabarcoding Sequencing for Laboratory-Raised Clonal Cultures
Source: Int J Environ Res Public Health. 2022 Apr 7;19(8):4446. doi: 10.3390/ijerph19084446 (PMC9024509; doi:10.3390/ijerph19084446)
Supplement: Supplementary file 1 [file ijerph-19-04446-s001.zip › Supplementary Figures.pdf]

Supplementary Figures

Supplementary Figure S1. Rarefaction curves of the 144 samples.

Supplementary Figure S2. Phylogram tree of the generated bacterial features at genus level.

Supplementary Figure S3. Heatmap analysis of bacterial communities based on top 30 most abundant features.

Supplementary Figure S4. Bar plot of significantly different bacterial phyla (a) and genera (b) between DINO and N-DINO groups.

Supplementary Figure S5. Prediction of the differential function of bacterial communities between DINO and N-DINO groups in KO assignments.

Supplementary Figure S6. Bar plot of significantly different bacterial genera between Thecate and Athecate groups.

Supplementary Figure S7. Alpha and beta diversity analysis between Thecate and Athecate groups.

Supplementary Figure S8. Prediction of the differential function of bacterial communities between Thecate and Athecate groups in KO assignments.

Supplementary Figure S9. Alpha and beta diversity analysis between Anti and N-Anti groups.

Supplementary Figure S10. Bubble plot for top 31 significantly different bacterial genera between Anti and N-Anti groups.

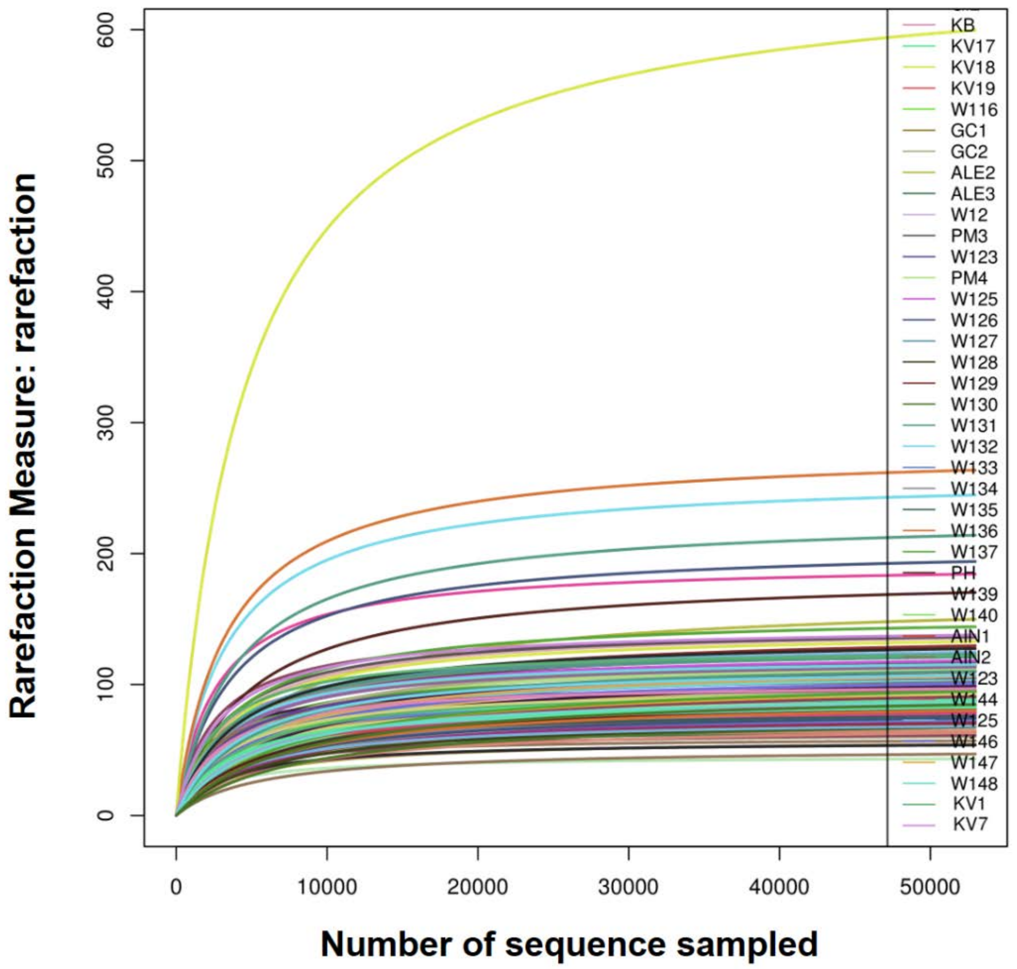

Supplementary Figure S1. Rarefaction curves of the 144 samples.

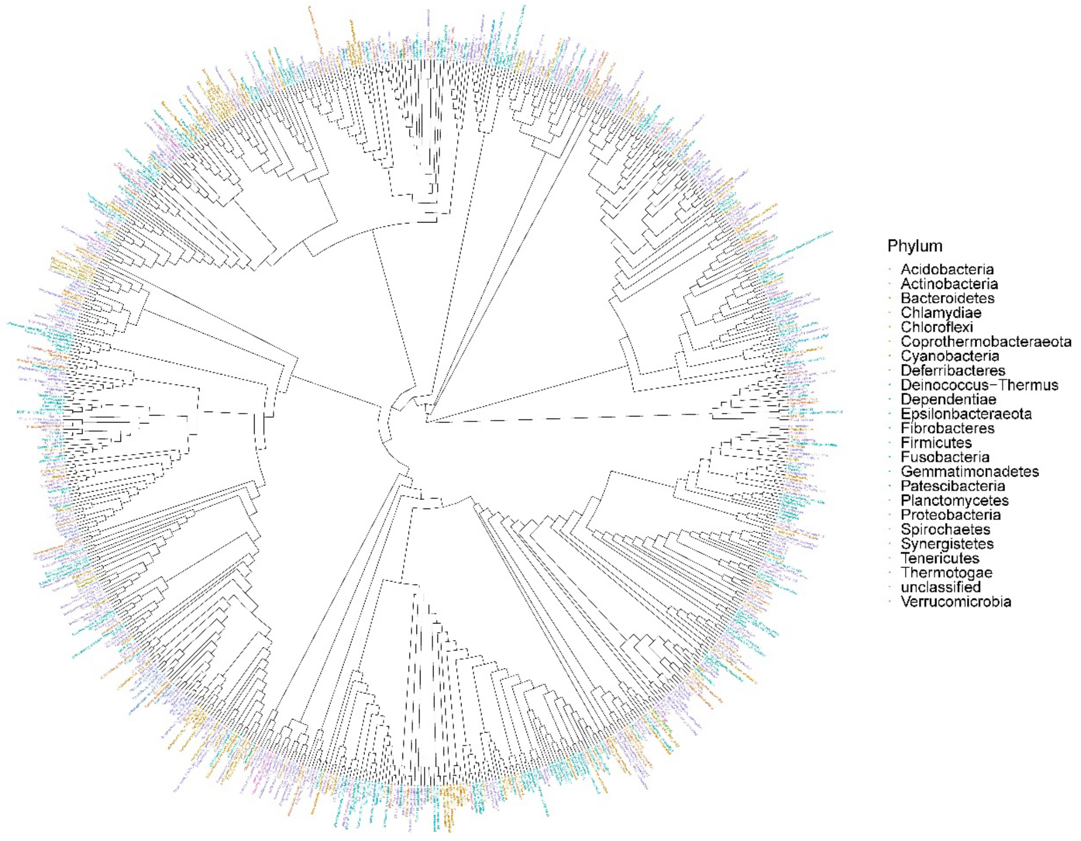

Supplementary Figure S2. Phylogram tree of the generated bacterial features at genus level.

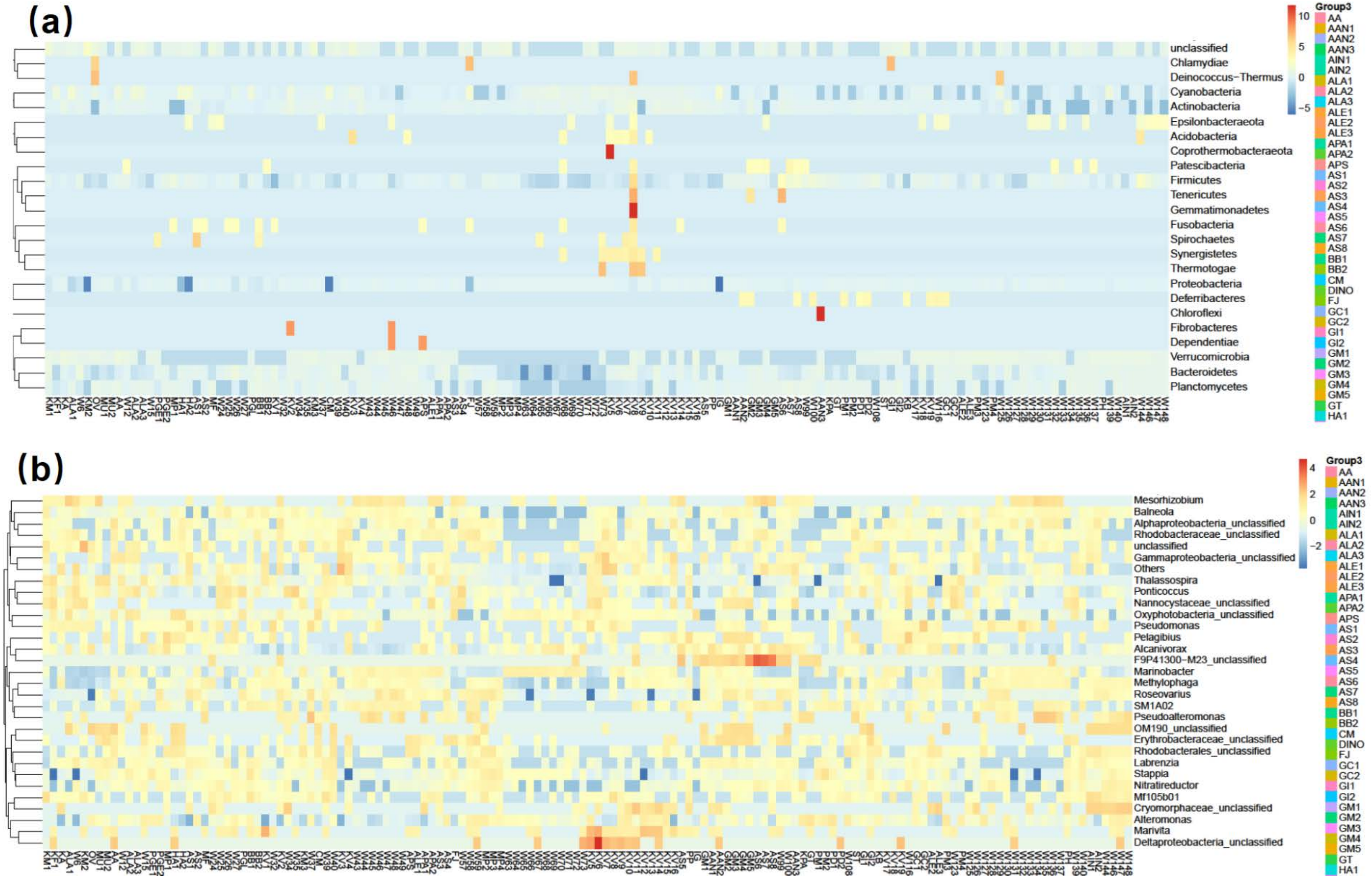

Supplementary Figure S3. Heatmap analysis of bacterial communities based on top 30 most abundant features at phyla (a) and genus (b) level.

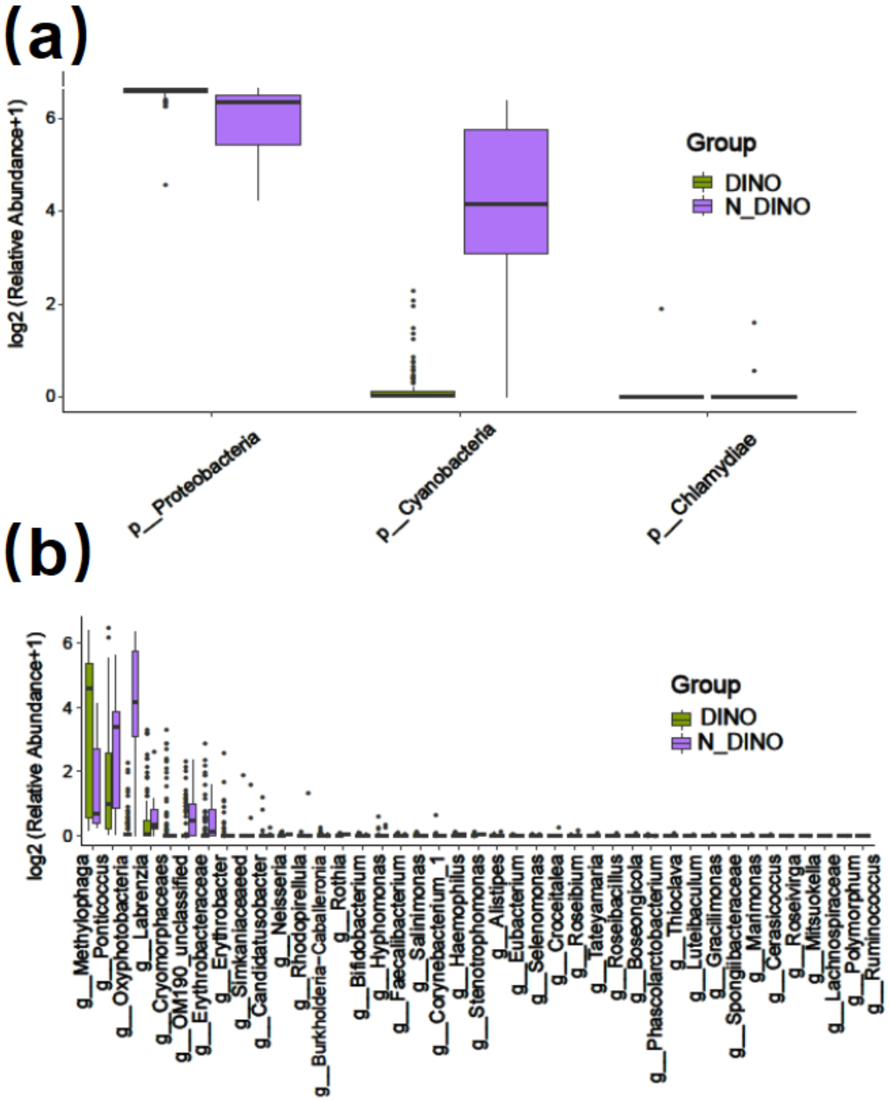

Supplementary Figure S4. Bar plot of significantly different bacterial phyla (a) and genera (b) between DINO and N-DINO groups (Fisher's exact test; $p<0.05$).

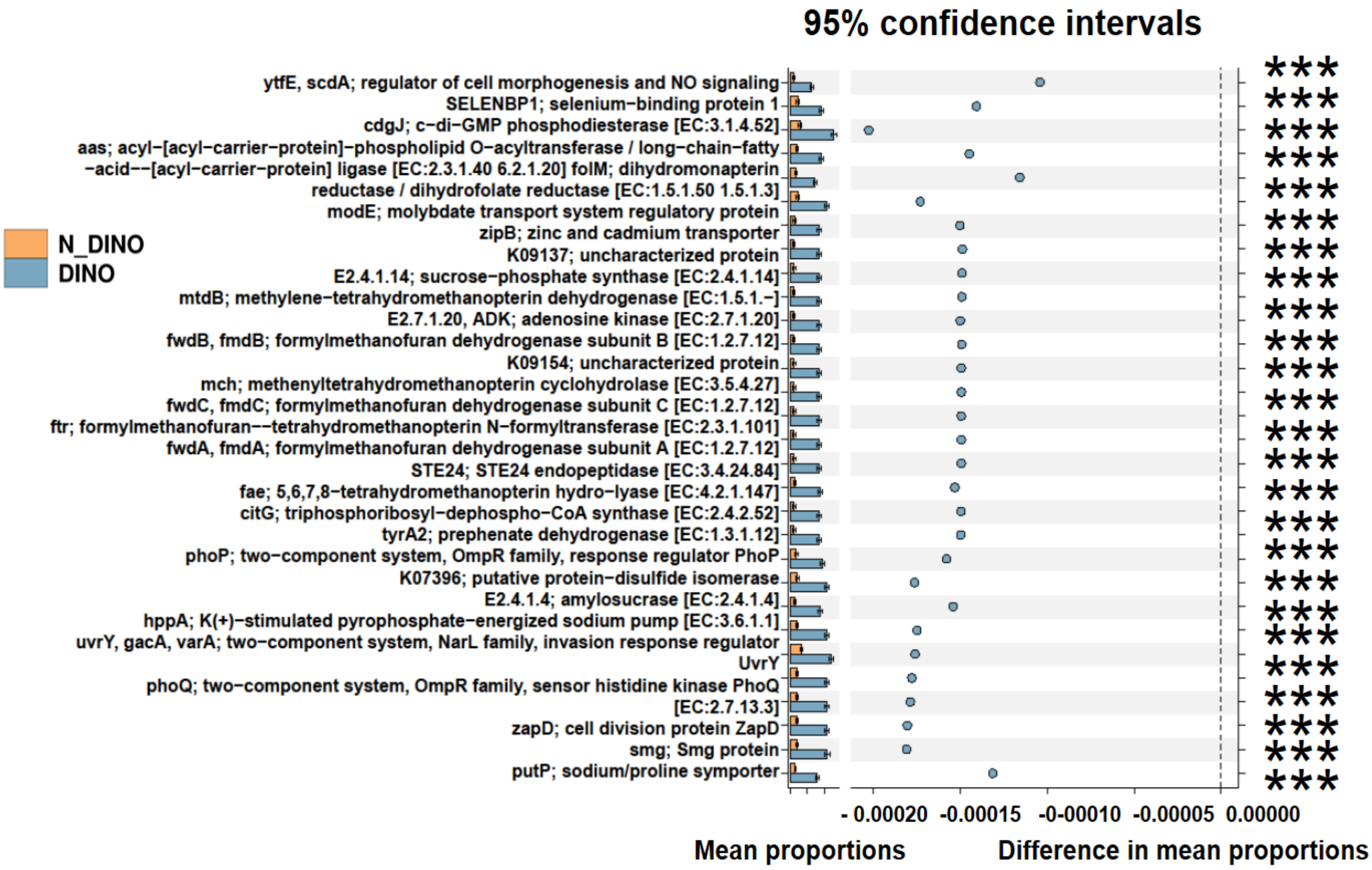

Supplementary Figure S5. Prediction of the differential function of bacterial communities between DINO (blue haze) and N-DINO (orange) groups in KO (KEGG Orthology) assignments. Gene functions were predicted from 16S rRNA gene-based microbial compositions using the PICRUSt algorithm to make inferences from KEGG annotated databases. Relative signal intensity was normalized by the number of the genes for each indicated metabolic pathway. *** indicates the difference is at a significant level with $p<0.001$.

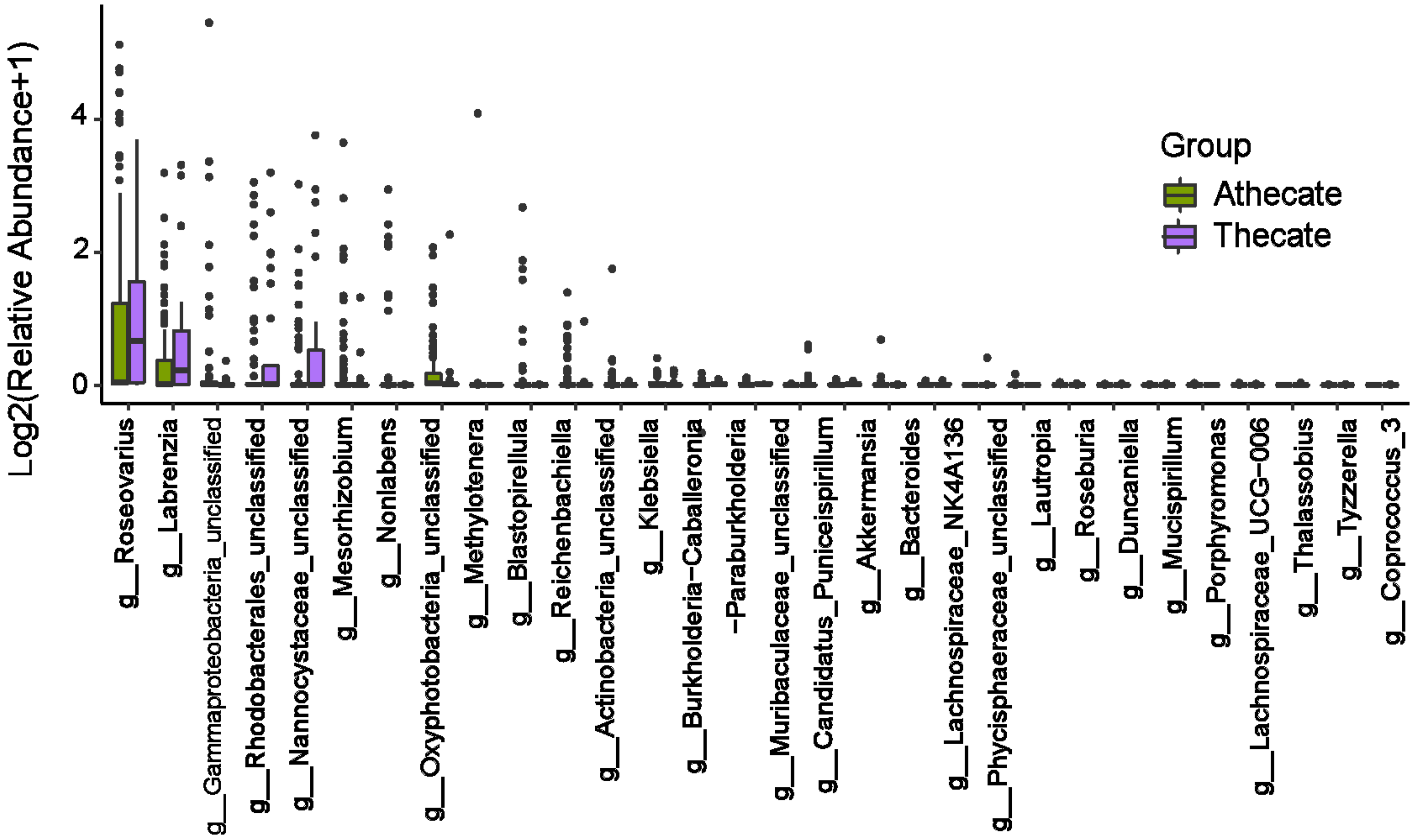

Supplementary Figure S6. Bar plot of significantly different bacterial genera between Thecate and Athecate groups.

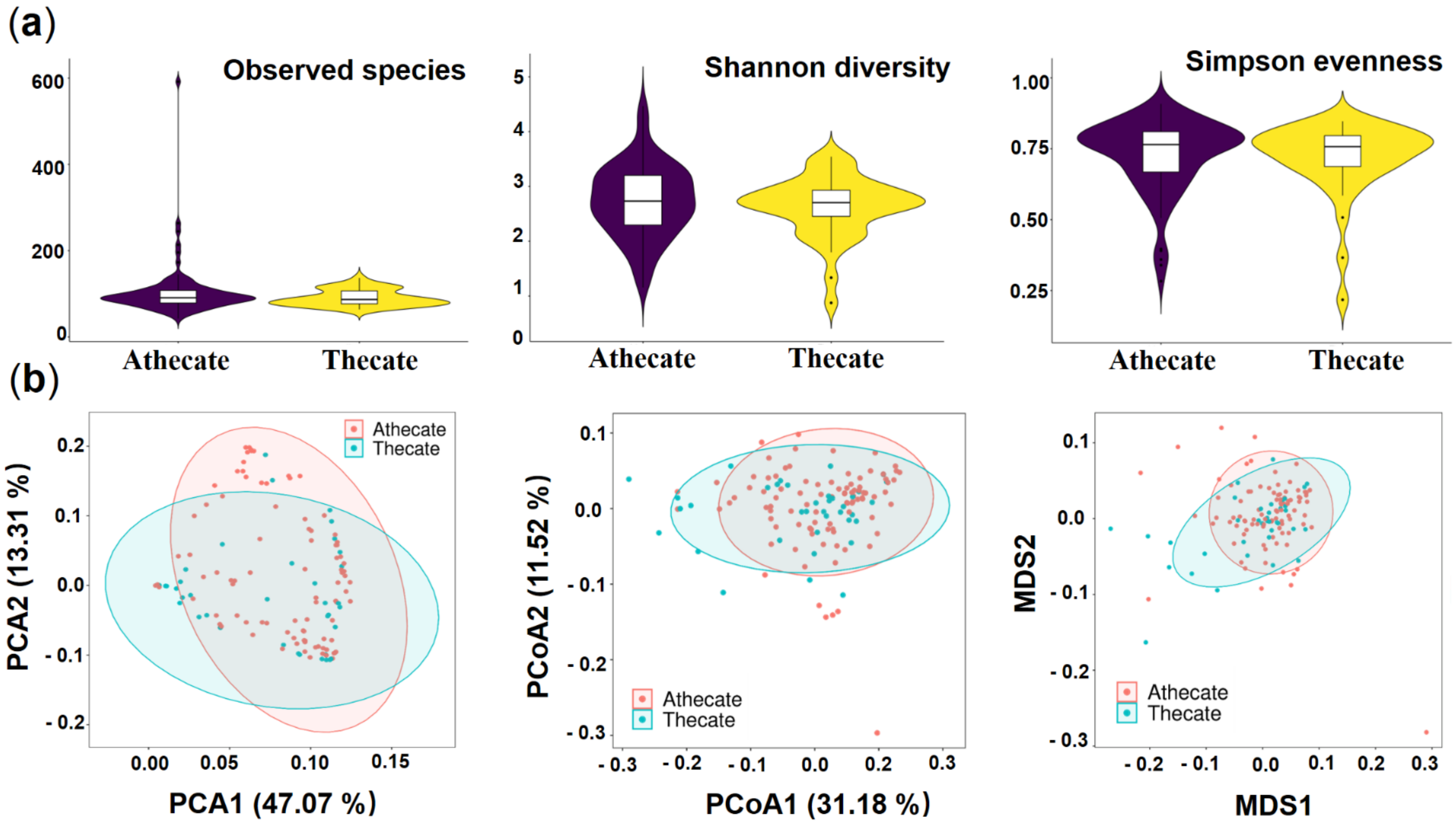

Supplementary Figure S7. Alpha (a) and beta diversity (b) analysis between Thecate and Athecate groups. (a) Violin plots (median, min and max) showing alpha diversity (Shannon diversity, Simpson evenness and the number of observed species) of Thecate and Athecate groups. (b) Beta diversity analysis of Thecate and Athecate groups. Principal component analysis (PCA) using genus/species-level Hellinger transformed relative abundances of bacterial sequences. Principal coordinate analysis (PCoA) using weighted-unifrac distances. Non-metric multidimensional scaling (NMDS) plot based on the weighted-unifrac distance using the Bray-Curtis similarity method.

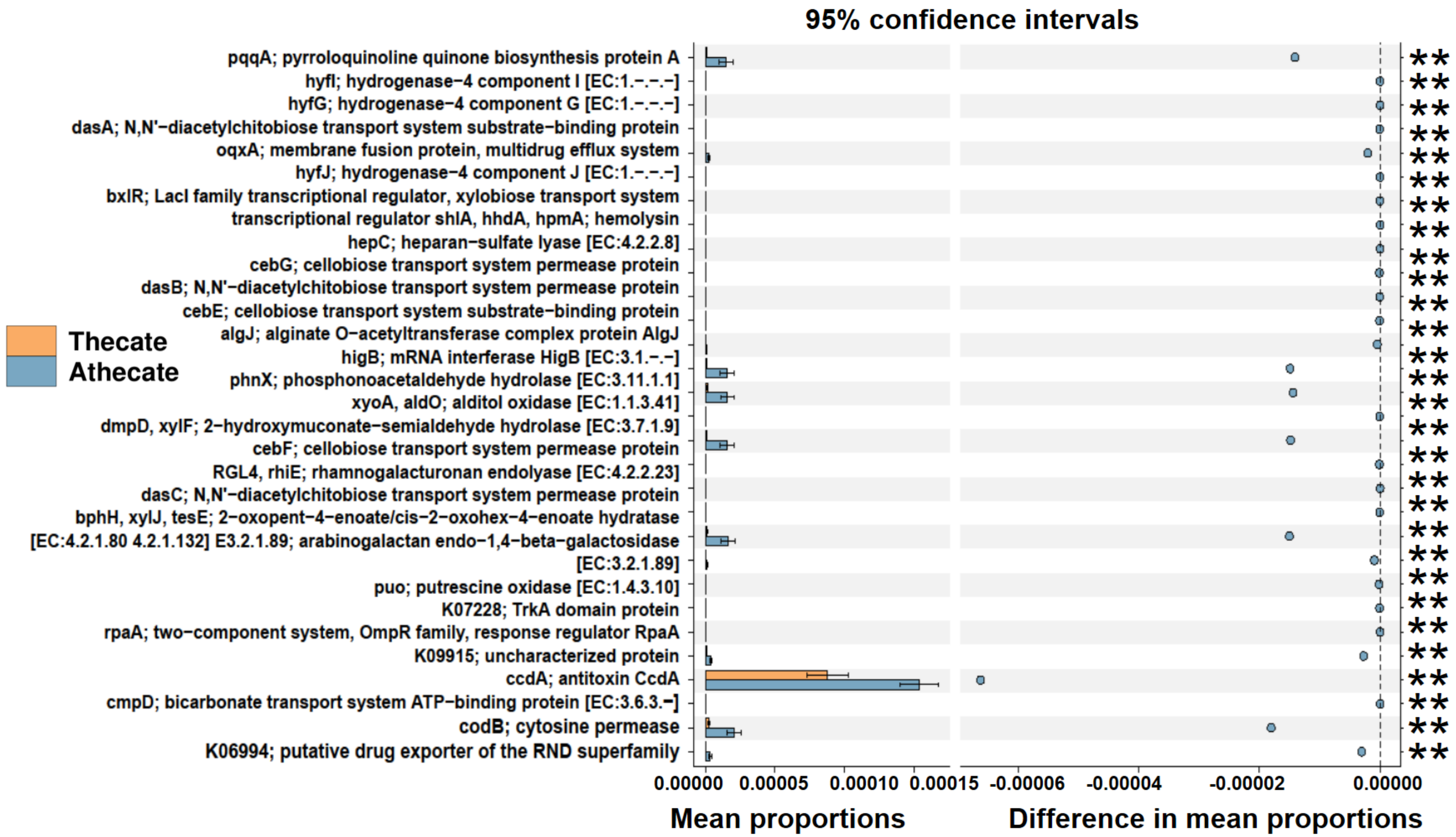

Supplementary Figure S8. Prediction of the differential function of bacterial communities between Thecate (orange) and Athecate (blue haze) groups in KO (KEGG Orthology) assignments. Gene functions were predicted from 16S rRNA gene-based microbial compositions using the PICRUSt algorithm to make inferences from KEGG annotated databases. Relative signal intensity was normalized by the number of the genes for each indicated metabolic pathway. ** indicates the difference is at a significant level with $p<0.01$.

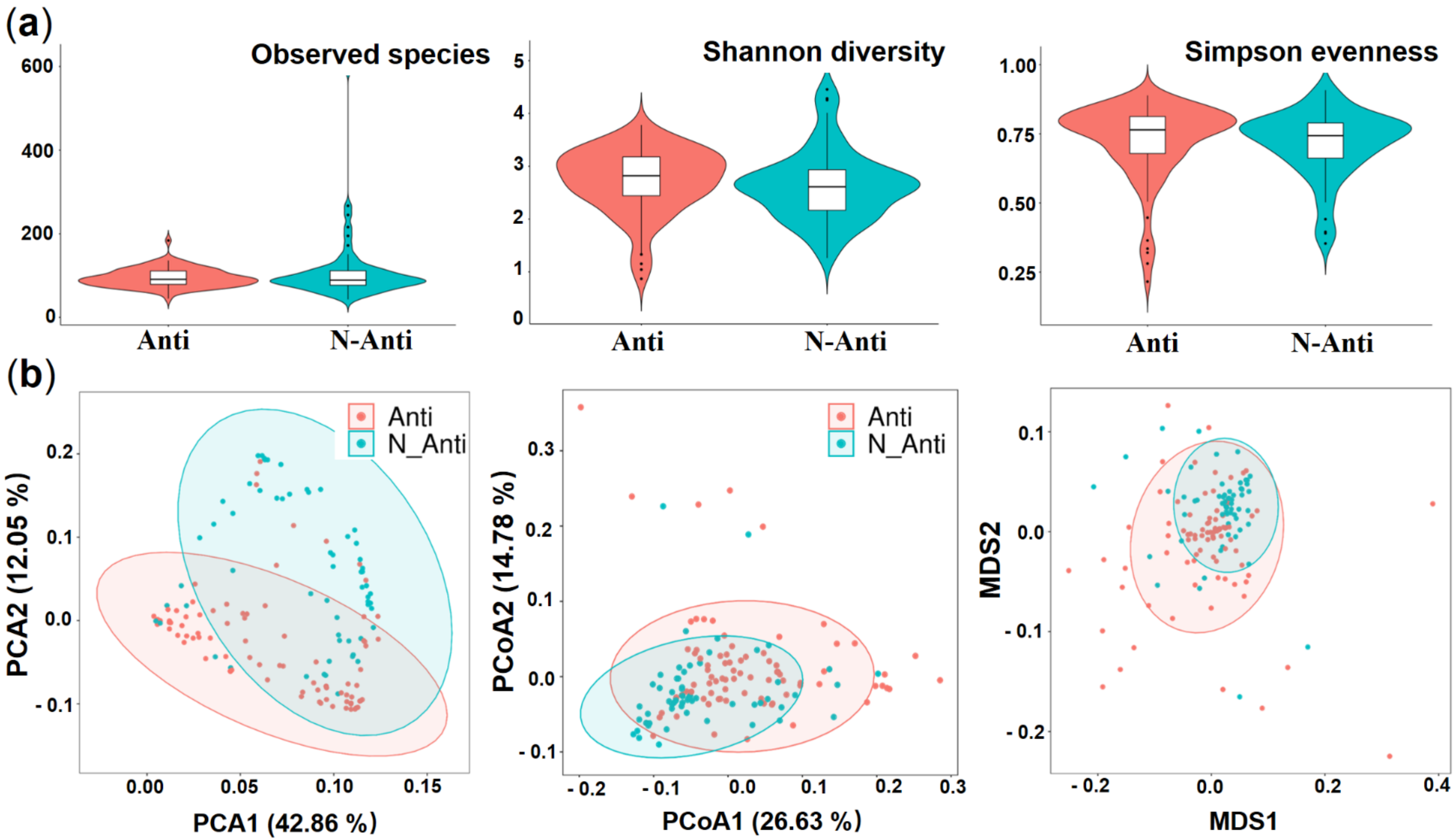

Supplementary Figure S9. Alpha (a) and beta diversity (b) analysis between Anti and N-Anti groups. (a) Violin plots (median, min and max) showing alpha diversity (Shannon diversity, Simpson evenness and the number of observed species) of Anti and N-Anti groups. (b) Beta diversity analysis of Anti and N-Anti groups. Principal component analysis (PCA) using genus/species-level Hellinger transformed relative abundances of bacterial sequences. Principal coordinate analysis (PCoA) using weighted-unifrac distances. Non-metric multidimensional scaling (NMDS) plot based on the weighted-unifrac distance using the Bray-Curtis similarity method.

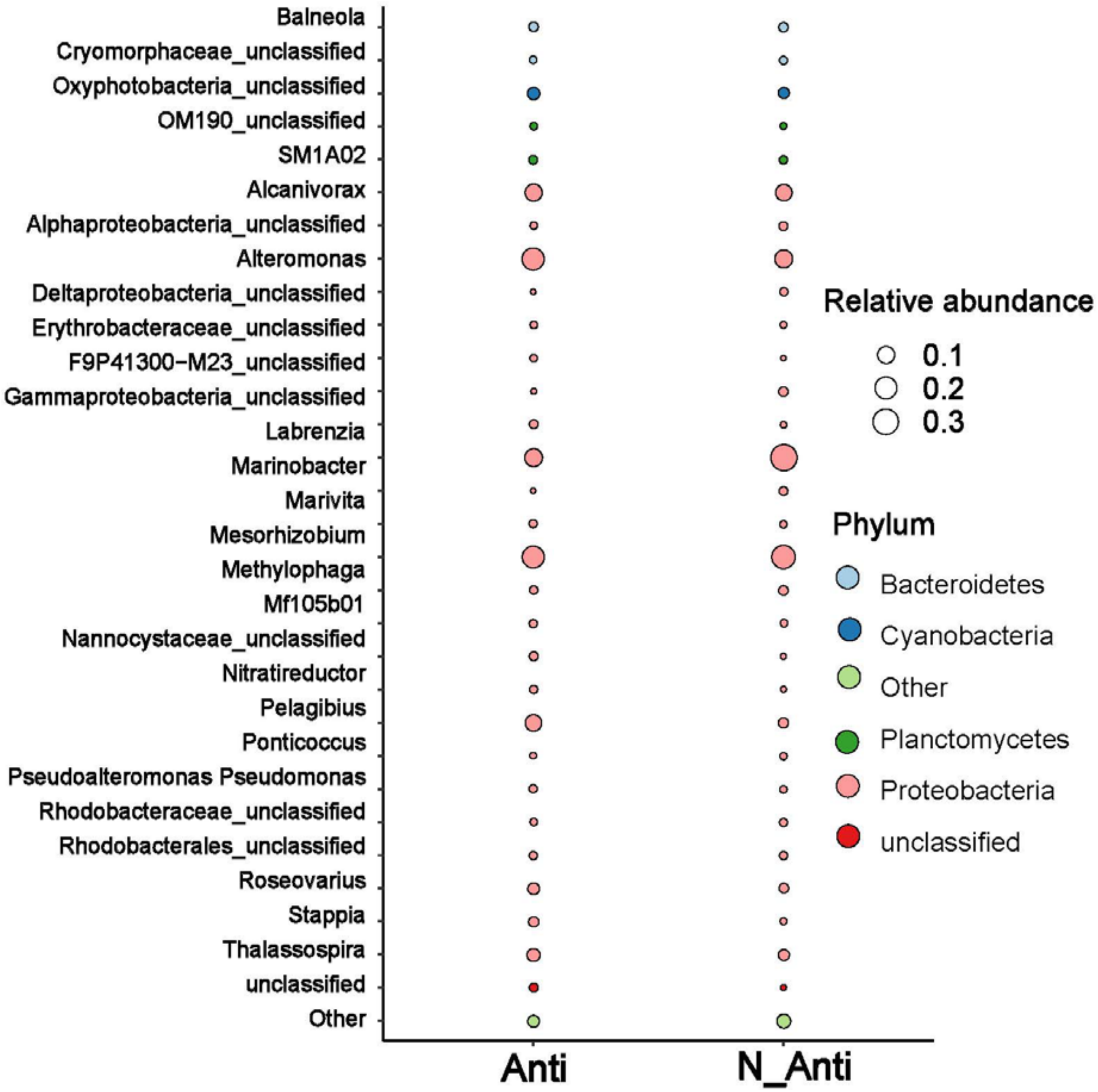

Supplementary Figure S10. Bubble plot for top 31 significantly different bacterial genera between Anti and N-Anti groups. The color of each dot represented different phyla. The size of dots represented the relative abundance of each genera.
